# Does Chemotherapy-Induced Gastrointestinal Mucositis Affect the Bioavailability and Efficacy of Anti-Infective Drugs?

**DOI:** 10.3390/biomedicines9101389

**Published:** 2021-10-04

**Authors:** Ana Rita da Silva Ferreira, Anne-Grete Märtson, Alyse de Boer, Hannah R. Wardill, Jan-Willem Alffenaar, Hermie J. M. Harmsen, Wim J. E. Tissing

**Affiliations:** 1 Department of Medical Microbiology and Infection Prevention, University Medical Center Groningen, NL-9713-GZ-1 Groningen, The Netherlands; a.r.da.silva.ferreira@umcg.nl (A.R.d.S.F.); a.e.de.boer.2@student.rug.nl (A.d.B.); 2 Department of Clinical Pharmacy and Pharmacology, University Medical Center Groningen, NL-9713-GZ-1 Groningen, The Netherlands; a.martson@umcg.nl; 3 Department of Pediatrics, The University of Groningen, University Medical Center Groningen, NL-9713-GZ-1 Groningen, The Netherlands; hannah.wardill@adelaide.edu.au (H.R.W.); w.j.e.tissing@umcg.nl (W.J.E.T.); 4 Adelaide Medical School, The University of Adelaide, Adelaide, SA 5005, Australia; 5 Precision Medicine (Cancer), South Australian Health and Medical Research Institute, Adelaide, NSW 5005, Australia; 6 School of Pharmacy, Faculty of Medicine and Health, University of Sydney, Sydney, NSW 2006, Australia; johannes.alffenaar@sydney.edu.au; 7 Westmead Hospital, Westmead, Sydney, NSW 2145, Australia; 8 Marie Bahshir Institute of Infectious Diseases and Biosecurity, University of Sydney, Sydney, NSW 2006, Australia; 9 Princes Maxima Centre for Pediatric Oncology, NL-3584-CS-25 Utrecht, The Netherlands

**Keywords:** cancer, chemotherapy, gastrointestinal mucositis, antibiotics, gut microbiota, drug pharmacokinetics

## Abstract

Antimicrobial prophylaxis is increasingly being used in patients with hematological malignancies receiving high-dose chemotherapy and hematopoietic stem cell transplantation (HSCT). However, few studies have focused on the potential impact of gastrointestinal mucositis (GI-M), a frequently observed side effect of chemotherapy in patients with cancer that affects the gastrointestinal microenvironment, on drug absorption. In this review, we discuss how chemotherapy leads to an overall loss of mucosal surface area and consequently to uncontrolled transport across the barrier. The barrier function is depending on intestinal luminal pH, intestinal motility, and diet. Another factor contributing to drug absorption is the gut microbiota, as it modulates the bioavailability of orally administrated drugs by altering the gastrointestinal properties. To better understand the complex interplay of factors in GI-M and drug absorption we suggest: (i) the longitudinal characterization of the impact of GI-M severity on drug exposure in patients, (ii) the development of tools to predict drug absorption, and (iii) strategies that allow the support of the gut microbiota. These studies will provide relevant data to better design strategies to reduce the severity and impact of GI-M in patients with cancer.

## 1. Introduction

Despite significant advances in the development of novel anti-cancer agents, chemotherapy remains the backbone of effective cancer control [1,2]. While highly effective, its use remains challenged by adverse complications, particularly when used in high doses [3,4]. High-dose chemotherapy is most frequently used to treat hematological malignancies, compromising the host’s immune cells prior to receiving a hematopoietic stem cell transplant (HSCT) [5,6]. Due to the severity of immunosuppression induced by this treatment, bloodstream infection is a common and potentially lethal complication. Approximately 20% of patients with hematopoietic malignancies for whom high dose chemotherapy is routinely used prior to HSCT develop bacteremia either as a result of exogenous contamination or the expansion and subsequent translocation of enteric pathogens across a compromised intestinal barrier [5,6,7,8]. In order to overcome these risks, anti-infective agents, including antibiotics, antivirals, and antifungals, are routinely used to control infection risk in vulnerable patient cohorts [9].

The efficacy of anti-infective agents relies on optimal intestinal function including absorption, transport, and metabolism [10]. However, due to the non-selective nature of chemotherapeutic compounds, healthy cells from the intestinal epithelium are targeted resulting in irreversible DNA damage and apoptotic cell death [11]. Consequently, the destruction of intestinal villi and the inability to rapidly repair the epithelial barrier during chemotherapy results in gastrointestinal mucositis (GI-M) [11,12,13]. GI-M is characterized by inflammation of the intestinal mucosa lining the gastrointestinal (GI) tract that leads to structural, functional, and immunological changes in the GI microenvironment [13]. Chemotherapeutic agents, commonly responsible for GI-M, are alkylating agents (busulfan, cyclophosphamide, cisplatin, melphalan), antimetabolites (5-fluouracil, methotrexate), topoisomerase I inhibitors (irinotecan), among others [14,15]. The exact mechanism of action of these agents and their corresponding impact on intestinal permeability are listed in Table 1. As these agents are often given in combination (e.g., FEC and FOLFOX), their toxicity is usually increased, which may worsen GI-M symptoms. Clinically, GI-M presents as ulcerative lesions, with associated abdominal pain, anorexia, and malnutrition [16]. In severe cases, GI-M can negatively impact anti-cancer therapy as often chemotherapy regimens have to be interrupted, which affects the treatment efficacy [7,17]. Although the incidence depends on the type of therapy and its dose, it has been estimated that close to 100% of people undergoing high-dose chemotherapy will experience GI-M [16,18,19,20].

GI-M pathobiology is currently proposed to involve five dynamic and overlapping phases [7,19,41,42,43]. Briefly, in the initiation phase, the penetration of chemotherapeutic agents from the submucosal blood supply induces direct DNA damage to the basal-epithelial cells, causing cellular stress and apoptosis. Consequently, the injured cells activate a variety of stress mechanisms which leads to the generation of reactive oxygen species as well as the release of pro-inflammatory cytokines. In the upregulation phase, these molecules act as highly effective secondary messengers and activate stress mechanisms in several mucosal-associated cells such as endothelial cells and macrophages. In turn, these cells respond by releasing a storm of pro-inflammatory cytokines, such as tumor-necrosis factor-α (TNF-α) and interleukin 1β (IL-1β), exacerbating tissue injury. During the third phase (signal amplification), the signaling mechanisms participate in a positive feedback loop whereby the original damage signals are amplified, thereby triggering the loss of self-renewal capabilities of epithelial stem cells and intensifying the state of inflammation. As a result, progression to the fourth stage (ulceration) commences whereby the integrity of epithelium is severely compromised, and frank ulceration occurs. It is in this stage that the symptoms and secondary complications of GI-M, including bacteremia, arise. Lastly, upon halting the chemotherapeutic intervention in the fifth stage, the mucosal barrier begins to spontaneously heal, inflammation subsides, and the mucosal barrier integrity begins to recover [19,41,42,43]. Ultimately, the profound epithelial damage observed during GI-M hampers one of the most important intestinal functions—the absorption of nutrients, and potentially drugs, across the GI tract.

The rate and degree of absorption of an orally administrated drug depend on several factors, including molecular size, solubility, degree of lipophilicity, and stability of the drug [10]. Together, these factors can have a great impact on the drug bioavailability and its transport across the absorptive epithelia [10]. Additionally, factors such as intestinal surface area, pH, blood flow, and intestinal motility can equally affect the absorption of a drug [44,45,46]. During chemotherapy, changes in the gastrointestinal microenvironment resulting from GI-M may therefore impact the key structures and functions required for drug absorption at multiple levels, thus resulting in alterations in systemic drug loads and efficacy [47].

Taken together, the complexity of the situation becomes clear: people with cancer are administered life-saving antimicrobials with no understanding of how GI-M impacts their bioavailability and thus efficacy. Despite this clinical paradox, few studies have been performed to understand whether such profound changes in the GI environment affect the absorption and efficacy of anti-infective therapies in the context of cancer treatment. In this review, we discuss how factors such as intestinal permeability, intestinal pH, and alterations in the composition of the gut microbiota may affect the absorption of anti-infective drugs.

## 2. Physiological Factors Contributing to Impaired Intestinal Absorption during Chemotherapy

**Figure 1:**
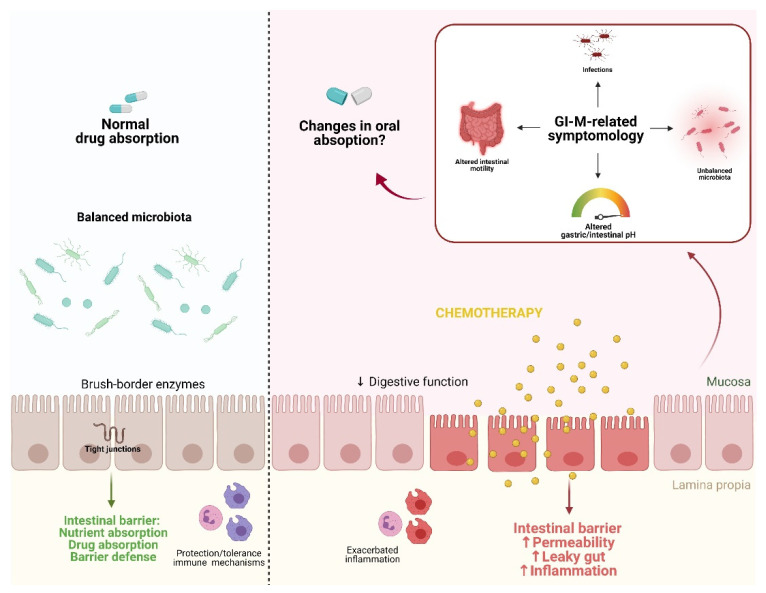
Proposed pathobiological aspects of GI-M contributing to changes in drug absorption. In a state of homeostasis, the combination of a balanced microbiota, tight junction formation, and balanced immune protection/tolerance mechanisms contribute to an optimal physiological function of the intestinal barrier, resulting in normal nutrient and drug absorption. The gastrointestinal microenvironment can be disturbed by external insults such as chemotherapy, leading to increased inflammation and consequently barrier disruption. This ultimately results in the disruption of the gut microbiota, alterations in gastric/intestinal pH, alterations of intestinal motility, and bacterial translocation. Together, these altered physiological/morphological functions potentially impair drug absorption. Figure created with Biorender.com.

The GI system is highly dynamic and organized, responsible for (i) separating the internal milieu of the outside environment and, (ii) digesting and absorbing nutrients [48,49]. Similar to nutrients, many orally-administrated drugs are also absorbed and metabolized in different parts of the GI tract (e.g., small intestine) [48]. For optimal absorption of drugs during chemotherapy, several assumptions are made about the GI microenvironment: (1) the intestinal architecture supports drug absorption, (2) factors such as intestinal pH and motility remain unaltered and thereby do not affect the bioavailability, activity, and toxicity of drugs, and (3) the gut microbiota remains unperturbed [50]. These assumptions are particularly relevant during GI-M as the GI environment is severely damaged. Figure 1 shows a graphical representation of proposed pathobiological aspects of GI-M contributing to changes in drug absorption.

### 2.1. Gastrointestinal Mucositis and Barrier Function

Intestinal atrophy and consequently impaired intestinal function are well-documented phenomena during GI-M [1,42,51,52,53]. This is supported by numerous studies showing that reduced citrulline levels (a non-essential amino acid synthesized by small bowel enterocytes and validated biomarker of GI-M) are associated with loss of enterocyte mass and consequently to a reduced mucosal surface area [54,55]. Reduced citrulline levels have also been associated with clinical observations of acute intestinal atrophy, undoubtedly suggesting that intestinal atrophy is linked to more severe clinical outcomes during GI-M [54,55]. As the intestinal villi are profoundly affected during GI-M, not only is the cumulative surface area of the small intestinal decreased, but also the activity of brush border enzymes. Brush-border enzymes are responsible for breaking down organic matter into absorbable molecules, and when damaged by chemotherapy, result in malabsorption [10,13]. In fact, Kuchay et al. 2015 showed that a single dose of 5-fluoruracil chemotherapy in rats is enough to cause a significant reduction in the activity of brush-border enzymes such as alkaline phosphatase, sucrase, and lactase, a result that needs to be reproduced in humans [56]. Ultimately, this demonstrates how chemotherapeutic drugs can negatively impact the normal architecture of the intestinal lining, consequently leading to an overall reduced capacity to absorb orally administered (antimicrobial) drugs.

In line with a reduction in surface area, the intestinal barrier is severely impacted during GI-M. The intestinal barrier is formed and maintained through the action of tight junctions, multi-protein complexes that partially contribute to maintain the intravascular volume and regulate the flux between vessels and organ parenchyma [57,58]. It is well documented that intestinal permeability is increased during GI-M, driven by proteolytic loss and internalization of key tight-junction proteins which render them ineffective [58,59,60,61]. Importantly, when the intestinal barrier is compromised, there is uncontrolled transit across the intestinal mucosa/epithelium. Given the sensitivity and degree by which the paracellular pathway is controlled in the intestine, this creates a very non-selective environment, potentially allowing orally administered compounds to cross into circulation with less resistance. This, in turn, could result in profound alterations in pharmacokinetic parameters including absorption, bioavailability, and organ distribution of drugs. More recently, other variables such as intestinal motility, luminal pH, and diet were suggested to contribute to impaired intestinal barrier function [38,62,63]. As such, an understanding of these variables is essential to understand their role in intestinal absorption.

### 2.2. Intestinal Motility, Luminal pH and Diet

Disturbances in the GI motility, frequently defined as diarrhea or constipation, are estimated to affect approximately 50–80% of cancer patients depending on the chemotherapy regimen [64]. Although diarrhea and constipation are well-recognized side-effects of anti-cancer treatment, very little research has been focused on their impact on intestinal absorption, particularly on drug absorption [64]. Animal studies have shown that destruction of the crypts of the small intestine by chemotherapeutic drugs resulted in metaplasia of goblet cells and excessive mucous secretion [64,65,66]. This, in turn, leads to a decreased absorptive capacity of the villi, thereby contributing to increased diarrhea and altered absorption [58,67]. Whilst diarrhea is considered to be largely the result of malabsorptive mechanisms (due to mucosal atrophy), evidence also suggests it is driven by hyper-motility in the gut [68]. In this case, there is a significant reduction in intestinal transit time, thus decreasing the exposure of orally administered drugs to the mucosa, thereby decreasing their bioavailability. This has been well documented in a review by Effingera et al. (2018). On the contrary, the frequent use of opioids in cancer patients may delay gastric emptying considerably, which may lead to an increased exposure of intestinal epithelial to drugs, possibly altering systemic drug concentrations [44,45,46,69]. Although evidence has suggested that alterations in intestinal motility may profoundly impact intestinal absorption, no studies have been yet performed in the setting of GI-M.

The release and absorption of many orally administrated drugs may also depend on the pH of the different sites of the GI tract. Although no evidence demonstrates consistent alterations of intestinal luminal pH during GI-M, anecdotal evidence suggests a shift towards a more alkaline environment due to decreases in bacterial metabolites, namely short-chain fatty acids (SCFA). SCFA are produced by commensal microbes in the gut and serve to (i) support epithelial growth and homeostasis and (ii) acidify the luminal environment to control enteric pathogens/pathobionts. It is well documented that the abundance of SCFA-producing microbes is decreased during GI-M, and decreases in SCFA have also been identified [35,51]. As such, it is likely that the luminal environment is more alkaline during GI-M, which may interfere with optimal drug dynamics. Such an alkaline environment was shown, for example, to decrease the bioavailability of posaconazole, which may hinder its efficacy [70]. Several clinical studies have reported pH-related changes in drug absorption observed in the setting of inflammatory bowel diseases (IBD) [71]. In a study by Fallingborg et al. (1993), six patients with active ulcerative colitis showed extremely acidic proximal colonic pH (ranging from 2.3 to 3.4) [62]. Similar results were observed by Sasaki et al. (1999) in four patients with Crohn’s disease, in which three presented lower right (pH 5.3) and left (pH 5.3) colonic luminal pH values, compared to normal controls (pH 6.8) [63]. Interestingly, the pH profiles in the proximal small intestine in twelve volunteers with IBD showed no significant differences compared to controls [71]. Unlike IBD patients where inflammation is usually lower in the GI tract, GI-M patients may suffer inflammation in the proximal small intestine. As a lower luminal pH is associated with intestinal structural changes, these results may suggest that luminal pH affects intestinal absorption and the correct delivery of different drug formulations [45,63].

Given the impact of diet on the GI microenvironment and the strong evidence that demonstrates altered dietary habits during GI-M, it is also critical that its influence on drug bioavailability be explored [72]. Diet plays a crucial role in intestine homeostasis, influencing digestion, the composition of the gut microbiota, and the function of the intestinal barrier [73]. For example, a high-fat diet can stimulate bile secretion, which interferes with the epithelial membrane and changes its permeability, thus increasing paracellular transport inclination and absorption [73]. On the other hand, a high-protein diet can inhibit specific intestinal amino/peptide transporters responsible for drug absorption, but also stimulate intestinal transporter systems and hepatic enzyme activity [69]. Studies have shown that the diet can also influence the absorption rate of orally administrated drugs [69]. For example, a full meal can increase the bioavailability of itraconazole, a frequently used antifungal agent, to a more significant extent than a light meal, while the intake of a high-protein diet can decrease the absorption of β-lactam drugs such as cephalexin and cefadroxil [74]. Interestingly, no differences in posaconazole exposure (tablets or capsule formulations) were observed between fed and fasted conditions in a study with 30 healthy volunteers [75]. In the context of high-dose chemotherapy, oral intake is severely decreased due to pain, nausea, and oral mucositis. This requires nutritional intervention, either in the form of enteral or parenteral nutrition. These approaches undoubtedly affect the microenvironment of the GI tract, however, have not been adequately explored for their potential to impact the bioavailability of orally administered drugs [76].

### 2.3. The Gut Microbiota

A growing body of research has suggested the fundamental role of the gut microbiota in the maintenance of intestinal homeostasis and gut resilience during chemotherapy [77,78]. Intestinal homeostasis is promoted in part by the secretion of microbial compounds by commensal bacteria that tightly control the multi-directional crosstalk of the gut microbiota, intestinal epithelia, and immune cells. Particularly, specific commensal bacteria are capable of suppressing inflammatory pathways, including the NF-κB pathway, and to induce the production of anti-inflammatory cytokines such as interleukin (IL)-10 [51,79]. Additionally, commensal bacteria also play an important role as regulators of intestinal permeability [51]. For example, bifidobacteria and lactobacilli have been shown to increase tight junction formation, which possibly contributes to normal intestinal barrier function [51]. Short-chain fatty acids, especially butyrate produced by bacteria such as *Faecalibacterium prausnitzii* and *Roseburia* spp., have been proposed to regulate intestinal permeability due to their ability to modulate epithelial cell viability [51,80]. Increased evidence has recognized the role of the gut microbiota on GI-M pathobiology, with alterations in the composition coinciding with the development of severe symptomology. Clinically, studies have reported that during GI-M a significant reduction in anaerobic bacteria (e.g., *Bacteroides, Lachnospiraceae (*formally called *Clostridium* cluster XIVa, *F. prausnitzii*, and *Bifidobacterium*) and an increase in *Enterococcus* spp. occurs. [16,51,81]. It is therefore not surprising that the reduction of commensal bacteria during the severe stages of GI-M may explain the increased intestinal permeability as a result of the exacerbated inflammatory responses, later contributing to malabsorption.

Evidence has shown that the gut microbiota can directly influence the bioavailability of oral drugs by affecting their metabolism, first-pass-effect, and enterohepatic metabolism [82]. This drug–gut microbiota effect is bidirectional as some drugs, including antibiotics, profoundly alter the composition of the gut microbiota. For example, Gu et al. (2020) investigated the short-term consequences of fluoroquinolone (levofloxacin) and β-lactam antibiotics (cefoperazone/sulbactam, and aztreonam) on the gut microbiota of mice [82]. In their study, the authors concluded that a 4-day treatment with β-lactams resulted in a significant reduction in butyrate-producing bacteria (*Roseburia*, *Lachnospiraceae*, and *Oscillospiraceae (formally called Ruminococcaceae*) and other beneficial taxa (*Blautia* and *Bifidobacterium*) [82]. Sulfasalazines (SSZ) are widely used to treat patients with IBD and rheumatoid arthritis. After oral administration, only 12% of SSZ was absorbed in the stomach and small intestine with the remaining drug being reduced by the gut microbiota to release 5-aminosalicylic acid (5-ASA), which is pharmacologically active. Therefore, as this drug should reach the colon to reduce intestinal inflammation, its bioavailability is profoundly influenced by the gut microbiota [83].

In the past few years, a new focus has been given to the impact of the gut microbiota on chemotherapy efficacy and toxicity [78,84,85]. In fact, several studies have shown that the efficacy of various conventional chemotherapeutics can be influenced by some specific microbiota. In a colon cancer mouse model, the intestinal enterobacteria were shown to influence the metabolism of the chemotherapeutic drug gemcitabine [84]. Similar results were observed by Iida et al. (2013) as the efficacy of oxaliplatin was attenuated in germ-free mice due to reduced intra-tumor reactive oxygen species generation [86]. Ultimately, these data explain the interactions between the gut microbiota and the host and how the process of tumorigenesis, as well as the efficacy of cancer treatment, are affected. Such interactions are well described in a “TIMER” mechanistic framework proposed by Alexander et al. (2017) [78]. Accordingly, the gut microbiota can modulate the efficacy of chemotherapeutic agents such as irinotecan, 5-FU, and methotrexate via TIMER: translocation, immunomodulation, metabolism, enzymatic degradation, and reduced diversity [78].

Antibiotics are able to profoundly alter the composition of the gut microbiota, which in turn, may positively or negatively affect the efficacy of cancer therapy [87]. These complex interactions were previously shown in patients with gastric cancer [87,88]. In fact, eradication of *Helicobacter pylori* with amoxicillin and clarithromycin in patients with early gastric cancer was associated with improvements in the grade of glandular atrophy at the corpus [88]. Ultimately, these results suggest that the modulation of the gut microbiota may influence not only the treatment outcome but also inflammation and carcinogenesis. As such, modulating the gut microbiota seems to be a promising strategy to restore intestinal homeostasis and therefore to optimize anti-cancer treatment.

## 3. The Effects of Gastrointestinal Mucositis on Drug Absorption

As previously discussed, several GI-M-related factors can potentially influence drug absorption [89,90,91,92,93]. However, the study of the impact of GI-M on drug absorption still remains a challenge, with only a few studies focused on a limited number of antimicrobial agents. In a cohort of 250 patients with haematological malignancies, of which 56 developed GI-M, Kovanda et al. (2017) concluded that mucositis had no influence on the bioavailability of isavuconazole (98.3% vs. 99.8%, non-mucositis vs. mucositis) [93]. The bioavailability of ciprofloxacin was previously studied, with different outcomes being reported. Gattis et al. (1997) observed no differences in exposure (at least not within 24 h after administration) between chemotherapy-induced grade I and II GI-M patients and healthy volunteers. In contrast, Johnson et al. (1990) showed an overall reduction in plasma ciprofloxacin concentrations (3.7 mg/L at 2–3 days post administration vs. 2 mg/L, 13 days after administration) in six patients diagnosed with GI-M [93,94]. Vanstraelen et al. (2016) investigated the pharmacokinetics of posaconazole dosing regimen in HSCT patients undergoing myeloablative or nonmyeloablative conditioning and found no clear correlation between plasma citrulline and plasma posaconazole [95].

Although insightful, these studies present several limitations, including their design and small sample size. As such, it becomes necessary to first perform high-quality studies in patients to better investigate and characterize the exposure of different orally administrated antimicrobial drugs. It is clearly not possible to predict the pattern of drug (mal)absorption for all drugs administrated to people undergoing intensive cancer therapy [46]. This demonstrates the need to develop models able to assess all physiological factors that contribute to drug absorption. A very insightful ex vivo model, increasingly used in several fields, is the Ussing chamber [96]. This new technique can be used to study bidirectional transepithelial drug transport in combination with intestinal metabolism. Moreover, the ability of the Ussing chamber to measure permeability quantitively makes it a useful tool to investigate how alterations in the intestinal architecture can impact drug availability [96]. Other in vitro systems such as gut-on-a-chip have been increasingly recognized for their controlled biochemical microenvironment thus supporting drug pharmacokinetic research [97].

More recently, a new mathematical modeling technique for predicting absorption, distribution, metabolism, and excretion of drugs has been developed. This is known as physiologically-based pharmacokinetics (PBPK) modeling [96]. This technique provides not only mechanistic insight into the physiologic and anatomic features of a drug, but also incorporates physiological variables of the host that may interfere with the efficacy of the drug [96]. The use of this modeling technique has been recently recommended by Pilmis et al. (2020) as they suggest that integration of PBPK modeling would be essential to interpret the impact of an orally administrated antibiotic on the different sites of the intestine and also on the gut microbiota [98]. The authors explain that along the GI tract, antibiotics are absorbed in a different manner, which suggests that if a drug is almost entirely absorbed in the small intestine, only a small portion will reach the distal digestive tract, resulting in a potentially high risk of infection [98]. Importantly, when combined with emerging epithelial modeling tools such as gut-on-a-chip, PBPK modeling can provide crucial information on drug absorption in the intestine [99].

These drug-absorption prediction techniques have not been yet applied in the study of the impact of GI-M in drug absorption. Therefore, before such techniques should be applied in the field, an effort should be made to design clinical longitudinal studies in people with varying degrees of GI-M to understand the dynamics of drug bioavailability during this common complication of cancer therapy. This will ultimately allow the delivery of a more personalized antimicrobial treatment to people with cancer, resulting in better infection control. It could even be argued that in the context of severe mucosal breakdown during GI-M, the transport of orally administered drugs into systemic circulation may in fact be increased. As such, restricted dosing of some antibiotics could be adopted in concerted stewardship initiatives to decrease rates of resistance.

## 4. Where Do We Go from Here?

Currently, the impact of GI-M on drug exposure is poorly understood. This is particularly concerning for cancer patients as they depend on anti-infectives to prevent potentially life-threatening infections. As such, efforts should be carried out to better understand the mechanisms that underpin drug absorption during GI-M. First, different clinical studies should be performed to assess the absorption of anti-infective drugs. These studies should include homogeneous cohorts and take into account variables including age, ethnicity, treatment modality, and genetic variants, as they may influence the drug’s bioavailability and efficacy. After the inclusion of more homogeneous groups, we recommend the determination of the plasma concentration of short half-life drugs (e.g., ciprofloxacin) in patients diagnosed with GI-M receiving chemotherapy. This will not only allow to determine the area under the concentration-time curve (AUC) but also possible associations between drug exposure and other patient-related factors. To investigate possible correlations between drug exposure and mucositis severity, we recommend the measurement of biomarkers of mucositis such as citrulline in patients’ blood plasma.

Secondly, we suggest that these clinical studies could be combined with the ex vivo and mathematical models previously discussed. The use of simple intestinal cell models cells (e.g., T84 and Caco-2) or more complex intestinal sections from rodents could provide crucial information including transepithelial drug transport, intestinal metabolism, and the regional differences in intestinal absorption. Moreover, as different tissues can be mounted in the Ussing chamber, human tissue derived from mucositis patients could be used to measure paracellular flux across the epithelium using fluorescent probes. This could provide real-time mechanistic insights into the impact of mucositis on drug transport across the epithelial barrier.

Lastly, restoring the gut microbiota homeostasis could potentially influence both tumorigenesis and consequently GI-M development. Therefore, we recommend the investigation of approaches such as probiotics, prebiotics, and fecal microbiota transplantation in the patients undergoing chemotherapy. Probiotic administration, for example, could restore microbial dysbiosis and maintain intestinal microbial balance by enhancing the gut barrier function and preventing colonization of pathogenic bacteria. Altogether, these clinical studies, in combination with innovative systems could provide us crucial information on drug exposure during different stages of GI-M. Unraveling the mechanisms beyond drug exposure of different anti-infectives used prophylactically during chemotherapy would help us to offer a better quality of life to cancer patients.

## 5. Conclusions

To date, the impact of GI-M on drug absorption has only been superficially addressed, with contradictory results. Here, we addressed the potential impact of GI changes on the absorption of drugs and reviewed the scarce literature studying this relationship. Given the lack of data, we recommend clinical studies to provide a direct correlation between GI-M pathobiology and drug exposure. Furthermore, we suggest the use of mathematical tools, intestinal cell and ex vivo models to study in detail the mechanisms underlying intestinal absorption.

## Figures and Tables

**Table 1:** Most common anti-cancer agents used during chemotherapy and impact on the gastrointestinal tract.

Class of Agent	Chemotherapeutic Agent	Mechanism of Action	Gastrointestinal Toxicity	Intestinal Permeability
Alkylating agents [14]	Busulfan [21] Cyclophosphamide [22] Cisplatin [23] Melphalan [24]	Cross-link between DNA/RNA strands	Mucosal ulceration ↑* Cell loss ↓^#^ Villus height ↑ Infiltrations [25,26]	Barrier disruption Bacterial translocation ↑ Permeability of the intestine (rats) [26]
Antimetabolites	5-Fluouracil [27,28] Methotrexate [29] Gemcitabine [30]	5-FU conversion to fluorouridine monophosphate (FUMP) Competitive inhibition of dihydrofolate reductase via displacement of dihydrofolate Incorporation of pyramidine analog into DNA	↑Inflammation ↑Crypt apoptosis ↑Villus atrophy ↑Increased risk of infection [31,32,33]	↑ Ratio of crypt cells to villous enterocytes ↑ Intestinal permeability (associated with reduced Zonula Occludens-1 expression in rats) [34] Bacterial translocation [35,36]
Topoisomerase I inhibitor	Irinotecan hydrochloride [37]	Inhibition of the DNA enzyme topoisomerase I	↑ Villus atrophy Crypt ablation Goblet cell metaplasia ↑Inflammation [19]	↓ Intestinal barrier function ↑ Intestinal permeability [38,39]
FEC	Fluorouracil, Epirubicin, and Cyclophosphamide	5-FU conversion to fluorouridine monophosphate (FUMP) + cross-link between DNA/RNA strands	↑ Paracellular permeability ↓ Intestinal barrier function [40]	↓ Glucagon-like peptide-2 circulating concentrations Mucosal ulcerations
FOLFOX	5-FU, leucovorin, and oxaliplatin	5-FU conversion to fluorouridine monophosphate (FUMP) + inhibits the synthesis of deoxyribonucleic acid (DNA)	↑Inflammation ↑Crypt apoptosis ↑Villus atrophy ↑Increased risk of infection [31,32,33]	↑ Ratio of crypt cells to villous enterocytes ↑ Permeability of the intestine (rats) [34] ↑ Intestinal permeability (associated with reduced Zonula Occludens-1 expression in rats) Bacterial translocation [35,36]

* ↑Increased; ^#^ ↓decrease.

## Data Availability

Not applicable.
